# Supplementary material for: Patterns of Intron Gain and Loss in Fungi
Source: PLoS Biol. 2004 Nov 30;2(12):e422. doi: 10.1371/journal.pbio.0020422 (PMC532390; doi:10.1371/journal.pbio.0020422)
Supplement: Table S1 — Also available at http://genes.mit.edu/NielsenEtAl/. (4.3 MB ZIP). [file pbio.0020422.st001.zip › NielsenEtAl/html/1441.html]

AN8870.1.NCU01452.1.MG06919.1.FG07480.1


```
 CLUSTAL W (1.82) Multiple Sequence Alignments - Introns Inserted


Sequence 1: NCU01452.1	337 aa
Sequence 2: FG07480.1	256 aa
Sequence 3: MG06919.1	256 aa
Sequence 4: AN8870.1	256 aa
Alignment Length: 337 aa
Number Identitical Residues: 207 aa
Alignment Score (without introns) 8240


MG06919.1 	------------------------------------------------------------
NCU01452.1	MNERVVQNLLQLAKTSSSEKKGACGLQSWKQKSPESNLPGNQGTQLWTRVEVATCHDKRY
FG07480.1 	------------------------------------------------------------
AN8870.1  	------------------------------------------------------------
          	                                                            

MG06919.1 	------------~------MAVGK2NKRLSKGKKGLKKKTQDPFARKDWYGIK0APAPFN
NCU01452.1	QLAPPCSALSPK0AQVLVKMAVGK2NKRLSKGKKGLKKKTQDPFARKDWYGIK0APAPFN
FG07480.1 	------------~------MAVGK2NKRLSKGKKGLKKKTVDPFSRKDWYSIK0APNPFN
AN8870.1  	------------~------MAVGK2NKRLSKGKKGIKKRTVDPFTRKDEYSVK0APSTFA
          	                   ***** **********:**:* ***:*** *.:* ** .* 

MG06919.1 	IRD2---VGKTLVNRSSGMKNANDALKGRIFEVSLADLQKDEDHAFRKIKLRVDEVQGKN
NCU01452.1	VRD2LFSVGKTLVNRTTGLKNANDALKGRIFEVSLADLQKDEDHSFRKVKLRVDEIQGKN
FG07480.1 	VRD2---VGKTLVNRTTGLKNANDALKGRILEVSLADLQKDEDHSFRKVRLRVDEVQGKN
AN8870.1  	NRD2---VGKTLVNRTSGLKNANDSLKGRIFEVSLADLQGDEDHAFRKVKLRVDEIQGKN
          	 **    ********::*:*****:*****:******** ****:***::*****:****

MG06919.1 	CLTNFHGLDFTSDKLRSLVRKWQSLIEANVTVKTTDDYLLRLFAIAFTKRRPNQIKKTTY
NCU01452.1	CLTNFHGLDFTSDKLRSLVRKWQTLIEANITVKTTDDYLLRLFAIAFTKRRPNQVKKTTY
FG07480.1 	CLTAFHGLDFTSDKLRSLVRKWQTLIEANVTVKTTDDYLIRLFAIGFTKRRGNQVKKTTY
AN8870.1  	CLTNFHGLDFTTDKLRSLVRKWQSLIEANVTVKTTDDYLLRLFAIAFTKRRPNQIKKTTY
          	*** *******:***********:*****:*********:*****.***** **:*****

MG06919.1 	AASSQIRAIRRKMTEIIQREASTCTLQQLTNKLIPEVIGREIEKATQGIYPLQN0VHIRK
NCU01452.1	AASSQIRAIRRKMTEIIQREASSCTLTQLTSKLIPEVIGREIEKATQGIYPLQN0VHIRK
FG07480.1 	AASSQIRAIRRKMTDIIQREASSCTLTQLTSKLIPEVIGREIEKSTQGIYPLQN0VHIRK
AN8870.1  	ARSSQIRAIRKKMTEIMQREASSCSLAQLTSKLIPEVIGREIEKATQGIYPLQH0VHIRK
          	* ********:***:*:*****:*:* ***.*************:********: *****

MG06919.1 	VKLLKQPKFDLGGLLALHGESTTDEQGQKVEREFKERVLEEV
NCU01452.1	VKLLKQPKFDLGALMSLHGESGSDEAGQKVEREFKETVLESV
FG07480.1 	VKLLKAPKFDLGALMALHGESGTDDQGQKVEREFKERVLEEV
AN8870.1  	VKLLKSPKFDLGALLNLHGESTTDDKGQKVEREFKEQVLESV
          	***** ******.*: ***** :*: ********** ***.*
```
